# Decadal radon cycles in a hot spring

**DOI:** 10.1038/s41598-017-12441-0

**Published:** 2017-09-21

**Authors:** Rui Yan, Heiko Woith, Rongjiang Wang, Guangcai Wang

**Affiliations:** 1 State Key Laboratory of Biogeology and Environmental Geology & MOE Key Laboratory of Groundwater Circulation and Environmental Evolution, China University of Geosciences, Beijing, 100083 China; 2 School of Water Resources and Environment, China University of Geosciences, Beijing, 100083 China; 3 China Earthquake Networks Center, 100045 Beijing, China; 4 GFZ German Research Centre for Geosciences, D-14473 Potsdam, Germany

## Abstract

A high-fidelity record covering nearly 40 years of water-dissolved radon from the hot spring site of BangLazhang (BLZ), Southwestern China is presented to study multi-year periodicities of radon. Ancillary observational data, i.e., water temperature, spring discharge rate, barometric pressure, combined with regional rainfall, galactic cosmic rays (GCR flux is modulated by solar wind and thus a proxy for solar activity) and regional seismicity from the same period are considered to identify potentially influencing factors controlling the changes in radon. Variations in radon concentration and ancillary observational data are studied using continuous Wavelet Power Spectrum (WPS), Wavelet Coherence (WTC), and Partial Wavelet Coherence (PWC). The results show that the long-period radon concentration is characterized by a quasi-decadal (8–11 years) cycle, matching well with the concurrent periodicity in water temperature, spring discharge rates and GCR. PWCs of radon, discharge rate and water temperature suggest that water temperature variations explain most of the coherent variability of radon and the discharge rate. We tentatively conclude that radon variations are mainly explained by variations in water temperature and spring discharge, which are modified and modulated by earthquakes and quasi-decadal variations of an unidentified process. The influence of solar activity on the decadal periodicity is discussed.

## Introduction

Radon (referring to^222^Rn throughout the text) is a naturally occurring radioactive noble gas with a half-life of 3.8 days. It is the decay product of radium-226 in the uranium-238 radioactive decay chain. Due to its radioactive decay, noble character, and omnipresence in nature, radon has found a wide range of applications in various fields of the geosciences^1^, from its frequent utilization as a potential earthquake precursor^2–6^, proxy for tectonic stress in volcanic environments^7,8^ and active tectonic zones^9^, to a wide range of applications as an environmental tracer in air, soil, marine and hydrological settings^8,10^.

Radon time series display complex dynamic characteristics, oscillating over multiple time scales^11–16^. Periodic radon variations are usually characterized by strongly non-stationary daily, multi-day, intra-seasonal and seasonal variability. Intra-seasonal to seasonal variations in radon concentration have been observed under different geological or meteorological conditions^11,17^.

Previous research indicates that variations in radon concentration are associated with a great number of influencing factors^18^. Meteorological conditions, such as atmospheric pressure^16,19,20^, temperature gradients^21^, rainfall^22^, and winds, are often thought to have a major impact. For example, Garavaglia, *et al*.^23^, and Zafrir, *et al*.^15^ found a precise correlation between the outside atmospheric temperature and radon concentration in an underground tunnel. They suggested that this temperature dependence is caused by convection currents in the tunnels induced by temperature differences between the tunnel and outside atmospheric air. Choubey, *et al*.^24^ found that the atmospheric temperature is positively correlated with radon emanation in a borehole in a seismically active area of the Garhwal region, northwest Himalaya. They suggested that the temperature dependence, facilitated by the temperature gradient in the borehole, controls the transport of radon from the deep interior to the surface. Recently, Zafrir, *et al*.^25^ developed a novel radon monitoring technique that enables the differentiation between seismo-tectonically-induced radon anomalies and diurnal and semidiurnal radon signals controlled by meteorological parameters. Perrier, *et al*.^26^ used a simplified mixing model to show that a slow infiltration corresponds to rich radon, which was used to explain the opposite seasonal variation at the summit and bottom of the Sur-Frêtes ridge (French Alps). Rock deformation and fracturing can release radon as demonstrated by laboratory experiments^27–29^. Besides the meteorological and environmental conditions, the possible influence of solar radiation originating in the deep solar interior was recently proposed^30,31^. Steinitz, *et al*.^31^ suggested that a component of solar irradiance is affecting the radiation from radon in air, and this influence is further modulated by the diurnal rotation of the Earth.

**Figure 1:**
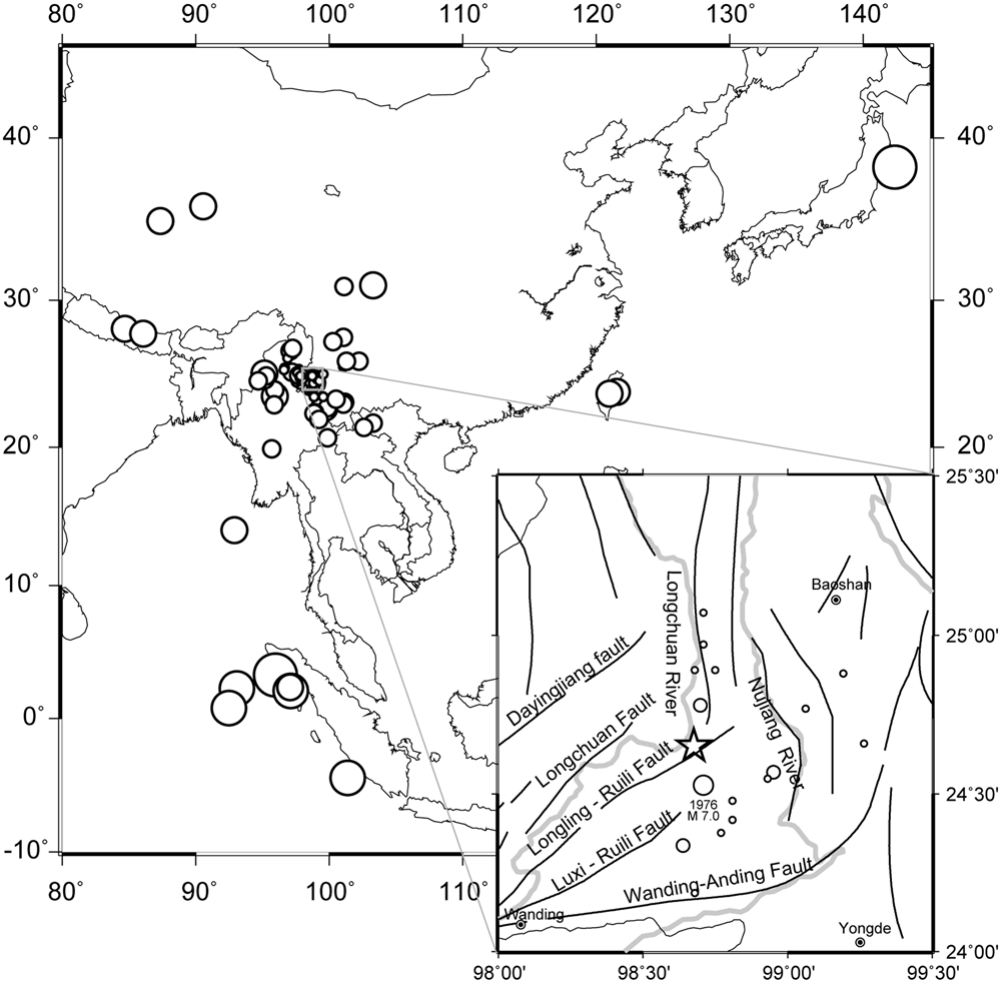
Location of the study area in SW China. The circles denote the earthquakes occurring between 1976 and 2015 with energy densities larger than 10^−3^J/m^3^ at the monitoring site (size of circles is proportional to magnitude of the earthquake). A zoom of the map shows the local tectonic setting and location of the BLZ hot spring site (star). Solid black and grey lines depict faults and rivers, respectively. This figure has been produced by using Generic Mapping Tools (GMT 4.5.2) software http://gmt.soest.hawaii.edu/.

Most of the previous studies about the periodical variation in radon are based on relatively short time series (up to several years at the most), and the reported periods of radon variation are mainly focused on diurnal to annual time scales. Interpretations as to the origin of the periodic signals often invoke either environmental (barometric pressure, air temperature, rain) variations or the influence of active geodynamic processes. In this work, a complete radon time series of nearly 40 years is presented, offering a unique opportunity to investigate long-periodic radon signals for the first time. Daily samples were taken at the BangLazhang (BLZ) hot spring, Yunnan province, SW China (Fig. 1, see details in the “Data” section). Here, we focus on the multi-year periodicity of radon and its potential influencing factors by using different kinds of wavelet transforms.

## Results

**Figure 2:**
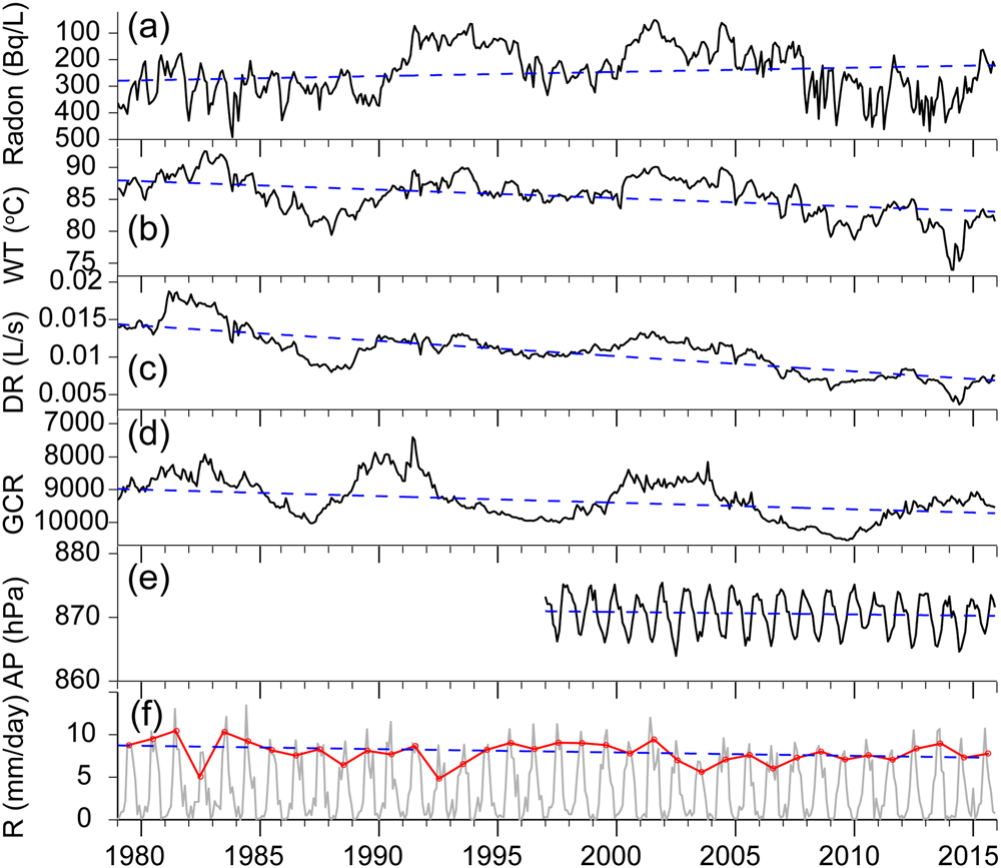
Time series of monthly groundwater parameters from 1979 to 2015 monitored at the BLZ hot spring site. The dashed lines indicate linear trends. From top to bottom panels: (**a**) Radon, (**b**) Water Temperature WT, (**c**) Discharge rate DR, (**d**) Galactic cosmic ray (counts/hour/100) GCR, (**e**) Atmospheric Pressure AP, (**f**) Rainfall R. Red line indicates monsoon-related rainfall (June, July, August). Note that the Y-axes are reversed for radon and GCR.

Due to a series of strong earthquakes in 1976 which occurred in close proximity to the monitoring site, radon, water temperature and spring discharge showed extremely large co- and post-seismic fluctuations (see supplementary materials). Thus, for the analysis of long-periodic variations, we excluded the first 2.5 years of monitoring data. Descriptive statistics of the measurements of radon concentration, water temperature and discharge rate for the time interval 1979 until 2015 are listed in Table S1. Monthly values of water radon, water temperature, discharge rate, GCR, atmosphere pressure and regional rainfall acquired at the BLZ hot spring site are displayed in Fig. 2. Radon, water temperature, and discharge rate show a weak decreasing trend over 40 years, whereas GCR increases over time (see linear regression lines in Fig. 2 and standard error of coefficient estimates in Table S2). Between 1979 and 2015, radon decreased from 280 to 210 Bq/L (−25%), water temperature dropped from 88 to 83 °C (−6%), and spring discharge decreased from 0.015 to 0.007 L/s (−53%).

Weak local maxima are superimposed on the general trends in the years 1982–1984, 1993–1994, 2000–2005, and 2012–2013. Similar patterns are visible in the radon time series – but less pronounced – with radon minima corresponding to maxima in water temperature and spring discharge. A visual inspection of the long-term trends suggests a positive correlation between water temperature and discharge (the correlation coefficients, taking into account that the two time series have significant auto-correlation, for monthly and annual averages are 0.86 and 0.91, respectively). Radon is positively correlated with GCR (0.38, 0.45), and negatively with water temperature (−0.74, −0.78) and discharge (−0.67, −0.73).

### Power spectrum densities (PSD)

**Figure 3:**
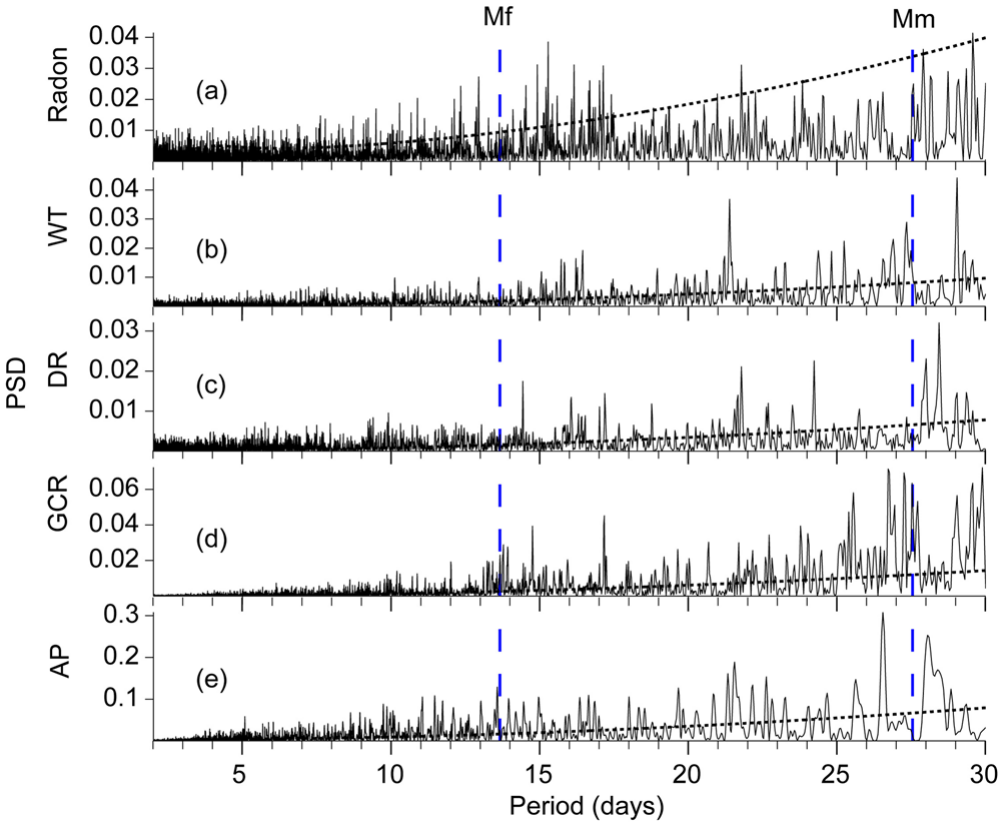
Spectra performed on de-trended and normalized data with a window size of 16,384 ( = 2^14^) days. A Hann window was used to reduce spectral leakages. (**a**) Radon, (**b**) Water Temperature WT, (**c**) Discharge rate DR, (**d**) GCR, (**e**) Atmospheric Pressure AP. Vertical dashed lines correspond to the Earth tidal bands M_f_ (13.66 days) and M_m_ (27.55 days). Dotted lines indicate 95% confidence levels.

In order to examine fortnightly to monthly variations, power spectrum densities were calculated with a window length of 16,384 ( = 2^14^) days. A Hann window was used to reduce spectral leakages (Fig. 3). The power spectrum density showed weak monthly variation in radon, water temperature and discharge rate. No indications for long-periodic tidal variations are apparent at about 13.66 days (corresponding to the tidal band M_f_ = 0.0732022 cpd) and 27.55 days (M_m_ = 0.0362916 cpd). A 27-day cycle displayed in the GCR is in agreement with the 27-day periodicity in solar activity (see e.g., Steinitz, *et al*.^31^ Shnirman, *et al*.^32^).

### Wavelet Power Spectrum (WPS)

**Figure 4:**
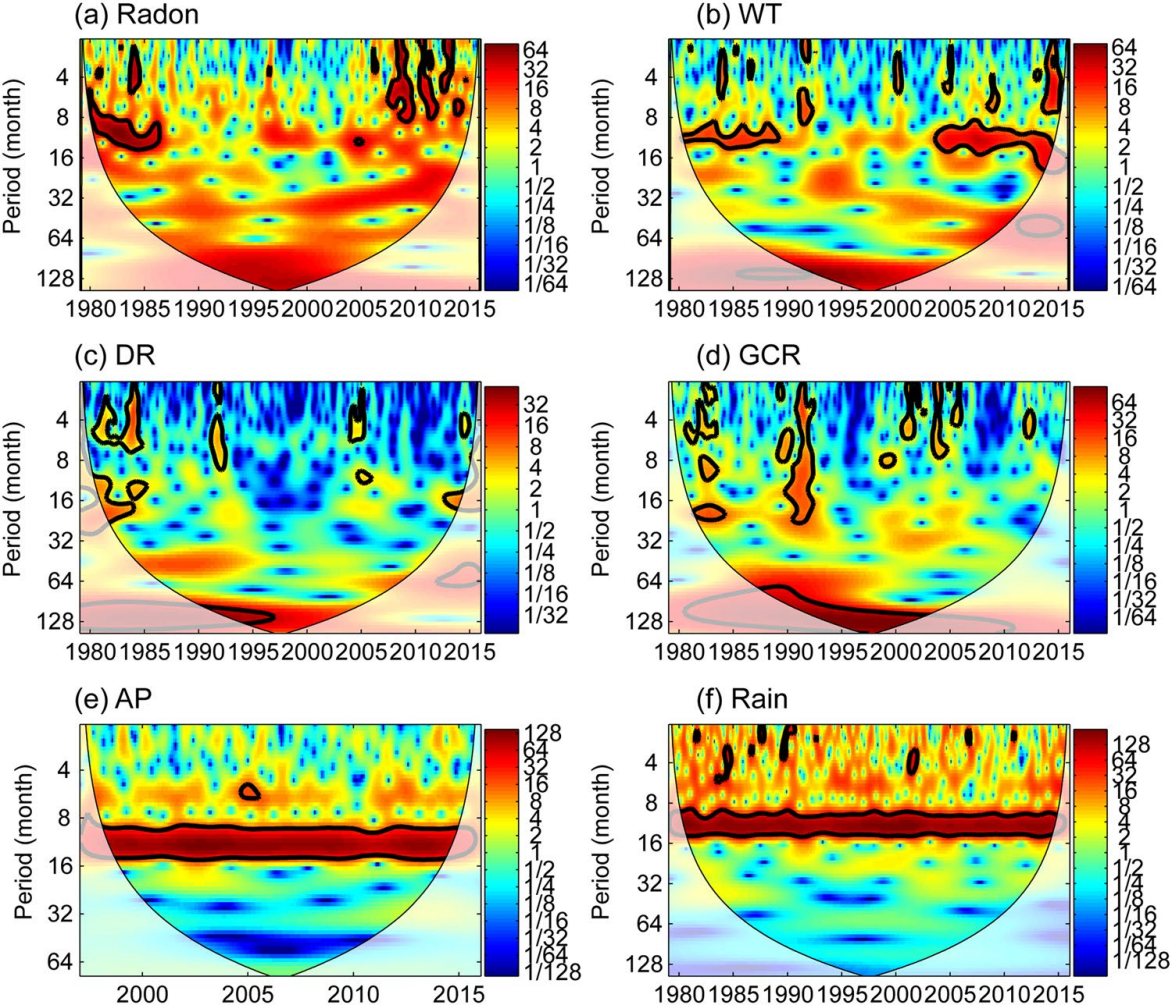
Wavelet power spectra WPS of each time series (1979–2015): (**a**) Radon, (**b**) Water Temperature WT, (**c**) Discharge rate DR, (**d**) GCR, (**e**) Atmosphere Pressure AP, (**f**) Rainfall. The thick black contour indicates the 95% confidence level and the lighter shade indicates regions inside the cone of influence (COI). The color scale in each panel corresponds to the amplitude of wavelet power spectra.

In order to identify oscillation periods, the method of wavelet power spectrum is applied to all time series. The mean and a linear trend are first removed from the time-series. Missing values (less than 0.33% of the data, the maximum gap length was 15 days) are filled by a linear interpolation before the calculation of the wavelet power spectrum. The results, shown in Fig. 4, indicate that there are common features in the wavelet power of the four time series of radon, water temperature, discharge rate and GCR, specifically in the band between 8 and 11 years. Discharge rate and GCR show the clearest signals (see dark red areas within the thick black lines indicating a 95% confidence level in Fig. 4). Barometric pressure and rainfall exhibit concurrently significant peaks in the annual band during the whole observation period, but do not show significant multi-year variability. In addition, the spectral powers of radon and water temperature also display a credible periodicity of one year.

### Wavelet Coherence (WTC)

**Figure 5:**
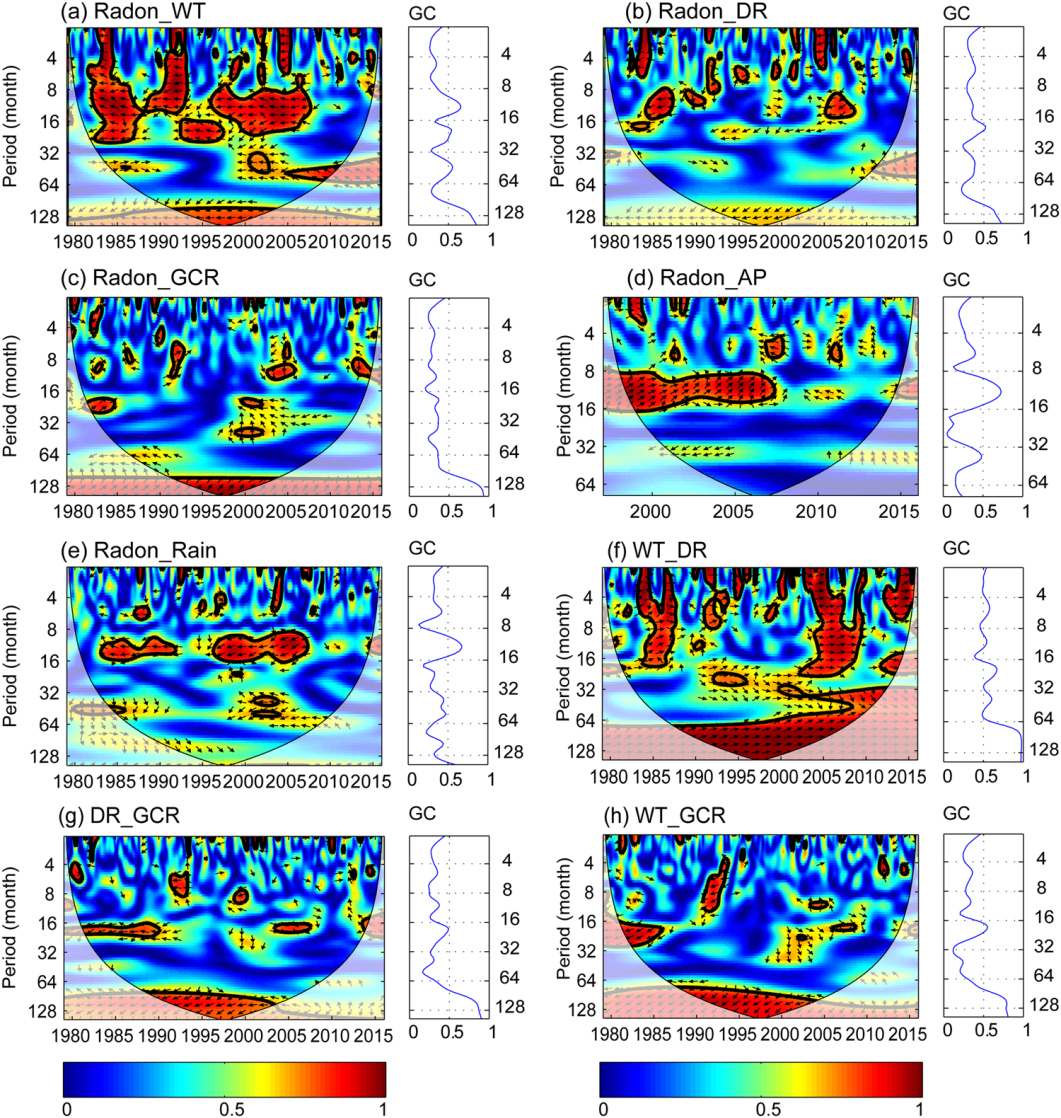
Wavelet coherences WTC (1979 to 2015) between (**a**) radon and water temperature, (**b**) radon and discharge rate, (**c**) radon and galactic cosmic ray, (**d**) radon and atmosphere pressure, (**e**) radon and rainfall, (**f**) water temperature and discharge, (**g**) discharge and GCR, and (**h**) water temperature and GCR. The thick black contour indicates the 95% confidence level and the lighter shade indicates those regions inside the cone of influence (COI). Arrows indicate the relative phase relationship (with in-phase pointing right, anti-phase pointing left, and phase-leading by 90° pointing straight down). Global coherence (GC) is shown for each sub-plot. Peaks of global coherence are listed in Table 1.

In order to examine the relationships between radon time series and potentially influencing factors, WTCs are calculated (Fig. 5). It is indicated that water temperature and GCR are highly coherent with radon at a >95% point-wise confidence level with coherence coefficients of >0.8 within the band between 8 and 11 years throughout the whole measurement period (Fig. 5a,c and Table 1). A similar, but slightly weaker correlation is evident for radon and water discharge (Fig. 5b). The mean phase angles between radon and water temperature are about 180 ° (arrows in Fig. 5a point towards the left). This anti-phase relationship matches well with the negative coherence coefficient found from the time series shown in Fig. 2. On the contrary, the phase angles between radon and GCR average at about 45° (see Fig. 5c), indicating a lead of GCR by about 1–1.5 years. Moreover, radon and water temperature show a high degree of coherence around the one-year band during the interval 1979–2008. Radon also shows some temporally localized coherence with water temperature, discharge rate and GCR below the one-year band. Rainfall and atmospheric pressure mainly contribute to radon at around annual periodicities. However, these coherences are not persistent. For example, within the significant region around the annual band, radon and atmospheric pressure only show a significant coherence during the interval of 1997–2007 (Fig. 5d), while radon and rainfall show a significant coherence during the interval of 1983–2007 (Fig. 5e).

**Table 1:** Peaks of global coherence coefficients. WT - Water Temperature WT, DR – Discharge Rate, GCR - Galactic Cosmic Ray, AP - Atmospheric Pressure.

time scale (year)	1	1.5	3.3	8–11
Radon_WT	0.66	0.54	0.55	0.85
Radon_DR	0.37	0.52	0.38	0.72
Radon_GCR	0.34	0.37	—	0.94
Radon_AP	0.73	—	0.48	—
Radon_Rain	0.67	—	0.45	0.60
WT_DR	0.54	0.67	0.61	0.97
DR_GCR	0.37	0.49	0.31	0.90
WT_GCR	0.34	0.55	0.24	0.81

Note: the time scales were obtained when the global coherence coefficients arrived at a local peak. When the time scale is between 8- to 11-year, global coherence coefficients are totally high. So the time scale of 8- to 11-year was selected as a significant time scale – indicates no local peak.

The highest coherence is observed between water temperature and discharge, which is already obvious from a visual inspection of the raw data. WTC between water temperature and discharge also shows a high coherence within the 8–11-year band. WTCs between discharge and GCR, as well as water temperature and GCR show a significant coherence in the 8–11-year band (Fig. 5g,h).

### Partial Wavelet Coherence (PWC)

**Figure 6:**
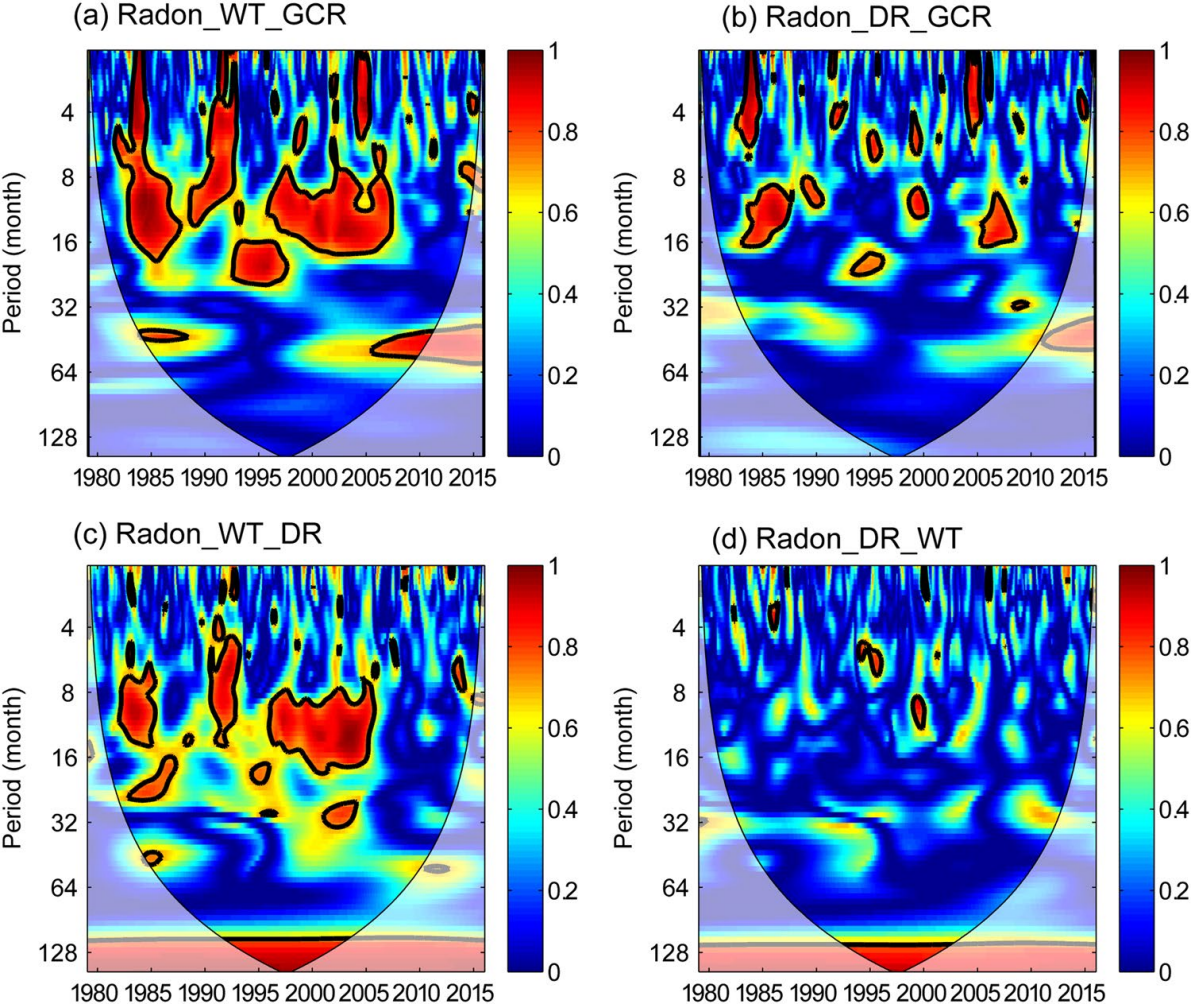
Partial wavelet coherences PWC among (**a**) radon, water temperature, and GCR, (**b**) radon, discharge rate, and GCR, (**c**) radon, water temperature, and discharge rate, and (**d**) radon, discharge rate, and water temperature. The thick black contour indicates the 95% confidence level and the lighter shade indicates regions inside the cone of influence (COI).

Since the time series of water temperature, discharge rate and GCR are all significantly correlated with radon within the 8–11-year band, the stand-alone relationship between radon and other influencing factors should be studied by removing the concurrent effect. PWC is a useful method to re-analyze the stand-alone coherence relationships between them. Figure 6 shows the PWCs of different combinations in terms of radon, water temperature, discharge rate, and GCR. The substantial disappearance in amplitude and areal extent of the previously found 8–11-year bands in the WTCs implies that the quasi-decadal periodicity observed in radon may be related to solar activity.

Moreover, the PWC of radon, water temperature and GCR (Fig. 6a) shows larger significant regions than that of radon, discharge rate and GCR at the whole band (Fig. 6b), which indicates that the effect of water temperature on radon is greater than the effect of discharge rate on water radon when the common effect of GCRs is conditioned out. Similarly, the PWC of radon, water temperature and discharge rate shows a larger significant region than that of radon, discharge rate and water temperature at the whole band (Fig. 6c,d), which indicates that the variability of water radon is highly coherent with water temperature. The reduction in significant regions of PWC of radon, discharge rate and temperature implies that there is a significant contribution of the water temperature to the relationship between radon and discharge rate. In other words, water temperature variations explain most of the coherent variability of radon and discharge rate.

## Discussion

The most obvious feature in the temporal pattern of long-term radon time series is a persistent trend with a quasi-decadal periodicity superimposed. Another obvious feature is an increase in radon volatility which started in 2007/2008 (see Figs 2 and S1). At the same time the correlation between radon and barometric pressure disappeared (Fig. 5d). To date, we have no explanation for these pattern changes. Continuous wavelet transforms applied in this work have allowed us to identify periodic variability in the time series of radon and its influencing factors. The WPSs of each time series, as well as the joint analysis using WTCs and PWCs, indicate that an obvious period of ~8–11-year exists in radon, water temperature, discharge rate, and GCR at the BLZ hot spring site. The nearly decadal periodicity of GCR is not surprising, as it is related to the ~11-year periodicity of solar activity^32–34^. However, how can decadal groundwater variations physically be related to the 11-year solar cycle?

Solar influence on winter severity had been claimed by Sirocko, *et al*.^35^, although Oldenborgh, *et al*.^36^ revealed convincingly that the claim was likely not justified. Other studies claim an influence of solar activity on streamflow^37^, the Pacific climate response system^33,38^, runoff^39^, groundwater recharge^40^, spring discharge^41^, and precipitation^42,43^, The latter authors analyzed 300 years of precipitation in the Huashan mountains in China and claimed that solar activity influences precipitation to some extent.

It is fair to note that solar influences on climate are complex and certainly difficult to access, as the reviews by Gray, *et al*.^44^ and Lockwood^45^ demonstrated. For solar cycle 23 Haigh, *et al*.^46^ could even show that the radiative forcing of surface climate by the Sun is out of phase with solar activity. Furthermore, volcanic eruptions contribute to climate variability^47^ and some authors claim to have identified periodicities close to the 11-year solar cycles^48^. The latter might be due to the observation that cooling of the atmosphere persisted for up to 10 years after major volcanic eruptions^49^. Although the coherence between radon and GCR is significant in the 8–11-year band in the wavelet domain, only a weak correlation exists in the time domain, which indicates the complexity of the system. After all, the radon time series corresponds to only ~3 samples of an ~11 years cycle. On the other hand, solar activity variations are not restricted to the 11-year-cycle, but appear in a suite of frequency bands. E.g. a 780-year annually resolved record of Indian Ocean monsoon precipitation (deduced from δ^18^O in speleothems) shows significant cycles at frequencies centered around 20, 16.1, 12.5, 6.6, and 4.5 years^50^.

It is important to note that a direct physical link between the solar (or volcanic) activity and the monitored groundwater parameters seems unlikely. Nevertheless, a link might be established via physical processes in the atmosphere, e.g. variations of cloud cover in response to cosmic rays^51^. The BLZ region is affected by the Indian monsoon and it could be shown that during the summer monsoon the extreme drought frequency is growing since 50 years, especially in the SW Yunnan province, where BLZ is located^52^. At first sight, the rainfall displays strong annual variations without any obvious long-term trend. But rainfall during the summer monsoon period (main wet season is in June, July, and August) shows a clear decreasing trend (Fig. 2f) which corresponds to the decreasing trend in spring discharge at BLZ hot spring. Interestingly, the authors claim annual drought periodicities of quasi-5-years and quasi-12-years.

Radon concentration are negatively correlated with the changes in water temperature and discharge rate. The PWC of radon, water temperature and discharge rate shows a larger significant region in the time-period plane than that of water radon, discharge rate and water temperature, which indicates that water temperature is a major influencing factor on both radon concentration and discharge rate at the BLZ spring site.

Physically, the dependence of radon on water temperature can be explained by the solubility coefficient *S* of radon in water, which is a function of water temperature. Between 1979 and 2015 the minimum and maximum temperature was 71 °C and 93 °C, respectively. Within this temperature range, the solubility differences are minor, i.e. less than 0.7% (see Supplementary Material, Fig. S5). Thus, temperature-dependent radon solubility is likely of minor importance at BLZ.

**Figure 7:**
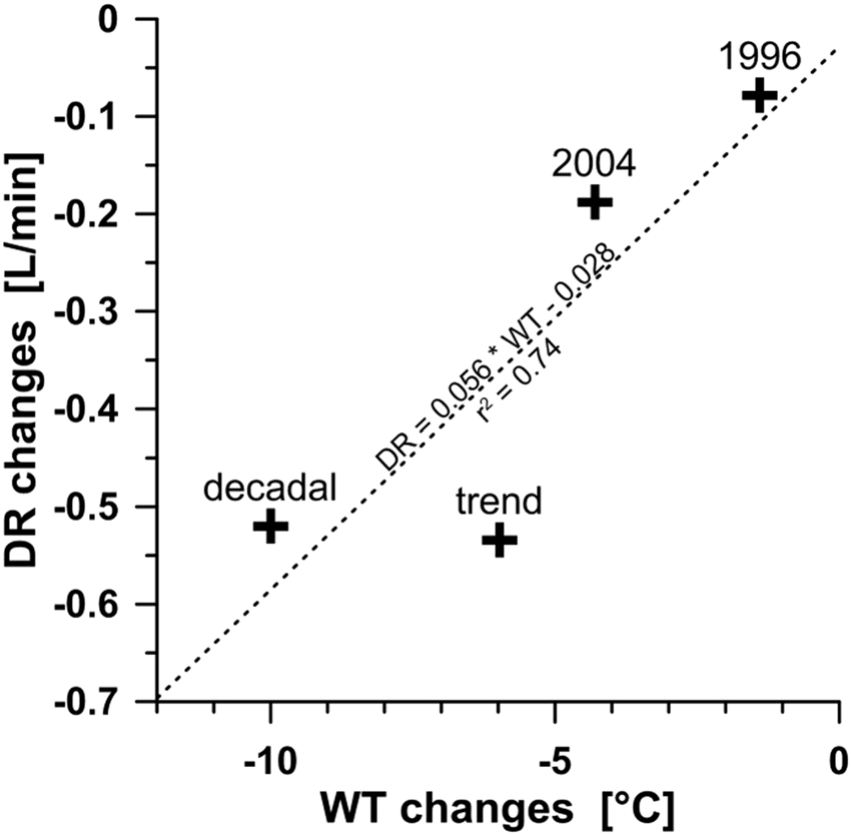
Relationship between changes in the discharge rate DR and water temperature WT resulting from co-seismic effects related to the earthquakes in 1996 and 2004 (see Table S3), the long-term trend, and the approximated amplitudes of decadal variations obtained from Fig. 2.

Wavelet analysis showed that (i) the highest coherence is observed between water temperature and discharge, and (ii) water temperature variations explain most of the coherent variability of radon and discharge rate. At BLZ, a temperature decrease of about 10 °C corresponds to a discharge reduction of about 0.5 L/min (Fig. 7). This relation could be established from a linear fit to observations covering different time scales, namely (i) the co-seismic response of the hydrogeological system to two earthquakes in 1996 and 2004, (ii) the long-term trend covering a time span of almost 40 years, as well as (iii) the approximate amplitudes of the quasi-decadal variations. Muraoka, *et al*.^53^ found a positive correlation between water temperature and discharge rate of hot springs in Japan, based on data from 3,686 sites. The authors assumed that the discharge rate of the hot springs is controlled by the buoyancy of hot water. For one-dimensional advection of heat in a porous media, the vertical Darcy velocity, ${\rm{\upsilon }}$, which is positive for down-flow, is expressed by the following equation according to the Turcotte and Schubert^54^:

1 $${\rm{\upsilon }}=-\frac{k}{\mu }{\alpha }_{f}{\rho }_{f0}g({T}_{r}-{T}_{0})$$

where $k$ is vertical permeability (m²), $\mu$ is fluid viscosity (Pa s) at the uniform reservoir temperature ${T}_{r}$ (K), ${\alpha }_{f}$ is the volume coefficient of thermal expansion of water (K^−1^), ${\rho }_{f0}$ is the density of water at the discharge temperature (kg/m³), $g$ is acceleration of gravity (m/s²), and ${T}_{0}$ is the surface temperature (K). We assume that µ = 1.01 × 10^−4^ Pa s, ${\alpha }_{f}$ = 10^−3^ K^−1^, ${\rho }_{f0}$ = 1000 kg/m³, $g$ = 9.80665 m/s², ${T}_{r}$ = 220 °C (from geothermometers), and ${T}_{0}$ = 290 K or 17 °C (annual average air temperature). If we further assume a total discharge DR = 15 L/s within an area A = 0.2 km² of the Banglazhang geothermal field^55^, a vertical velocity υ = DR/A is given, and thus eq.(1) can be solved for the unknown permeability. A vertical permeability *k* = 3.8 × 10^−15^ m² is thus obtained. A value of 3.0 × 10^−16^ m² would be obtained after taking mixing into account. Li, *et al*.^56^ estimated that the BLZ hot water is a mixture of 8% deep water and 92% shallow groundwater. Thus, the discharge rate of the deep fluid coming from the reservoir would only be DR = 1.2 L/s (8% of 15 L/s). Temperature-wise, the mixing model of Li, *et al*.^56^ would require mix of 8% reservoir fluid at 220 °C and 92% of shallow groundwater of 70 °C to obtain the observed surface temperature of 82.6 °C. Usually, shallow groundwater shows the average annual air temperature, here 17 °C. Thus, either the estimated mixing ratio is wrong, or another mixing member is needed to explain the observations at the BLZ geothermal field. Given the uncertainties, in the following considerations, we use a permeability of 1.0 × 10^−15^ m² ( = 10 mDarcy), which seems to be a reasonable value when compared to other geothermal fields. Björbsson and Bodvarsson^57^ compiled a global data set with permeability values ranging from 1 to 100 mDarcy.

We now assess the hypothesis that the long-term decreasing discharge at BLZ is possibly caused by the cooling of the reservoir. The permeability is fixed at 10^−15^ m² and all other parameters are the same as above. First, the discharge area for a single spring is set to 760 m² to obtain DR = 0.9 L/min, which was the average discharge in 1979 (see linear regressions in Fig. 2). Second, the reservoir temperature is reduced until DR = 0.42 L/min is obtained, the average discharge value measured at the end of 2015. To achieve this, a reduction of reservoir temperature from 220 °C to almost 160 °C would be required, corresponding to a cooling rate of 1.5 °C/a. Similar cooling rates were found at the Wairakei geothermal reservoir in New Zealand^58^. But, contrary to BLZ, the Wairakei reservoir is heavily exploited including re-injection of cold water. Furthermore, there are no indications for reservoir cooling based on geothermometers. Thus, we reject the cooling hypothesis.

For short-term variations like the co-seismic temperature and discharge changes, a different explanation is required anyway. Groundwater flow is very sensitive to permeability changes. Reducing the vertical permeability in eq. (1) from *k* = 1.0 × 10^−15^ m² to *k* = 0.9 × 10^−15^ m², results in a decrease in the discharge rate by 0.1 L/min, which has been observed co-seismically at BLZ (see Figs S2–S 4). Earthquake-induced permeability changes of the order of 1–5 × 10^−15^ m² had been repeatedly observed at two wells in California^59^.

It should be noted, that the numbers given above are first-order estimates at best, because the underlying assumption of fluid flow through a porous medium is likely not to be justified due to the existence of fault zones. Furthermore, the mixing model of BLZ has obviously to be improved. Relationships between discharge rate and water temperature may be explained by a pressure-driven control on the mixing ratio of the different water types^60^. The discharge changes due to changes in the velocity caused by either pressure variations at depth or by changes of the cleft width. An increased velocity would bring more hot deep thermal fluids to the surface, increasing water temperature, and vice versa. If we assume that radon solubility in deep thermal water is lower than that in the shallow cold water, the radon concentration in the deep thermal water is likely to be less than that of the shallow water. Thus, radon is negatively correlated with both, water temperature, and discharge rate.

In tectonically active areas, radon concentration and other hydrogeological parameters may correlate with seismic activity in the near and far field^6,61–63^, aseismic movements of active faults^64^, or pre-slip prior to earthquakes (precursor)^65^. At the BLZ hot spring, co- and postseismic hydrogeological changes induced by earthquakes were found. The most obvious changes in hydrogeological data sets at the BLZ hot spring site were observed after the 1976 Longling *M*_W_ 7.0 earthquake and the 2004 Sumatra *M*_W_ 9.1 earthquake. Figures S2 to S4 show the response and slow recovery to the pre-earthquake values, indicating that hydrogeological parameters recovered to background levels within one year. Similar recovery times were observed repeatedly by Wang, *et al*.^66^ at an artesian well in response to distant earthquakes. It could be demonstrated that the mixing ratio between shallow and deep fluid components changed co-seismically, and recovered to pre-event ratios within several months after the event^67^. Thus, the relatively short recovery periods suggest that the 8–11-year variations in radon and temperature cannot be explained by a single earthquake, but would need multiple seismic events which is not supported by data (Fig. S1d).

## Methods

### Wavelet transform

This technique is used to decompose the time series into a time-frequency space with multi-time resolution. It is a powerful tool for analyzing nonstationary time series with different power^68,69^. There are two kinds of wavelet transform, the continuous wavelet transform (CWT) and the discrete wavelet transform (DWT). The latter has been applied to identify changes in the pattern of diurnal and semi-diurnal variations of radon monitored at a hot spring in Tiberias, Israel^70^. In this work, the data are analyzed based on CWT. The techniques of continuous wavelet power spectrum, continuous wavelet coherence and partial wavelet coherence are used to examine the periodicity and dependence of radon on some possible influence factors. The Morlet wavelet is selected since it provides a good balance between time and frequency localization. Monte Carlo methods are used to determine the statistical significance level of WPS, WTC and PWC^71^. The Cone of Influence (COI) is used to evaluate the edge effects caused by discontinuities at end points^69,72,73^.

### WPS (Wavelet Power Spectrum)

The continuous wavelet transform (CWT) projects a time series onto a wavelet function, which is time-frequency space localized and has zero mean. For a time series (${x}_{n}$, *n* = 1, …, *N*) with uniform time steps $\delta t$ and a specific wavelet function $\psi (x)$, the CWT can be easily defined as the convolution of ${x}_{n}$ with the scaled and normalized wavelet:

2 $${W}_{n}^{X}(s)=\sqrt{\frac{\delta t}{s}}\sum _{{n}^{^{\prime} }=1}^{N}{x}_{n\text{'}}\psi \,[(n\text{'}-n)\frac{\delta t}{s}]$$

where *s* is the wavelet scale^73^. The calculations may be done more quickly by implementing *N* times the convolution for each scale in Fourier space^69^. WPS is defined as ${2}^{j}\times {|{W}_{n}^{X}(s)|}^{2}$, where *j* is the scale level and ${2}^{j}$ is multiplied to correct the bias towards low-frequency oscillations^72^.

### WTC (Wavelet Coherence)

WTC is an enhanced tool for identifying possible relationships between two processes by searching frequency bands and time intervals during which they covary^72–74^. Similarly to the definition of the traditional correlation coefficient, WTC squared of two time series $x$ and $y$ can simply be defined, as

3 $${R}^{2}(x,y)=\frac{{|S({s}^{-1}W(x,y))|}^{2}}{S({s}^{-1}W(x))\cdot S({s}^{-1}W(y))}$$

where the $W$ operator is the continuous wavelet transform when it has one argument and the cross-wavelet transform when it has two, $S$ is a smoothing operator, and $s$ is wavelet scale. The term “coherence” usually stands for the WTC squared, which ranges from 0 to 1. The same applies for the PWC discussed in the following section.

### GC (Global Coherence)

The global wavelet coherence coefficient at a certain scale, *s*, is defined as time-averaged wavelet coherence coefficients,

4 $${\bar{R}}^{2}(s)=\frac{1}{N}\sum _{\tau =1}^{N}{R}^{2}(s,\tau )$$

where $\tau$ is the location parameter in the time domain, *N* is the number of point in the time sereis^69^.

### PWC (Partial Wavelet Coherence)

PWC is a technique similar to partial correlation that helps to identify the resulting WTC between two time series y and x _1_ after eliminating the common influence of time series x _2_. Following Mihanović, *et al*.^75^ and Ng and Chan^72^, PWC squared can be defined by an equation similar to the traditional partial correlation squared, expressed as

5 $$R{P}^{2}(y,{x}_{1},{x}_{2})=\frac{{|R(y,{x}_{1})-R(y,{x}_{2})\cdot R{({x}_{2},{x}_{1})}^{\ast }|}^{2}}{{[1-R(y,{x}_{2})]}^{2}{[1-R({x}_{2},{x}_{1})]}^{2}}$$

where $R$ operator is WTC, $R(x,y)=\sqrt{{R}^{2}(x,y)}$.

In terms of PWC, a low ${{RP}}^{2}(y,{x}_{1}{,x}_{2})$ implies that the time series ${x}_{1}$ does not have a significant influence on the time series $y$ over a particular time–frequency space, and time series ${x}_{2}$ is the dominate effect on the variance of $y$. If both ${{RP}}^{2}(y,{x}_{1}{,x}_{2})$ and ${{RP}}^{2}(y,{x}_{2}{,x}_{1})$ have significant bands, both ${x}_{1}$ and ${x}_{2}$ have a significant influence on $y$. Usually, PWC is combined with WTC to identify the common dependence of multiple time series.

## Data

### Hydrothermal area of BangLazhang

The BangLazhang (BLZ) hot spring (98.68 °E, 24.65 °N, 1,280 m above sea level) is located in Yunnan Province, SW China, within the hydrothermal zone of Longling city (Fig. 1). The climate is of a subtropical monsoon-type with a mean annual air temperature of about 17 °C. Annual rainfall amounts to 1,500 mm. The Xiangbai River is the major surface water source, running through the Banglazhang spring area, flowing from east to west and discharging into the Longchuan River. The hydrothermal area consists of several sets of hot spring clusters with more than 100 hot springs. To date, no boreholes have been drilled in the BLZ hydrothermal area. Based on detailed field investigation and hydrogeological analysis of the hot spring cluster area, the No. 1 hot spring had been designated as a continuously hydro-chemical monitoring site with the aim of detecting earthquake precursors. The outlet of the hot spring is situated in a concave calcified granitic pool with a depth of 2 m and a diameter of 3 m. Previous studies of hydrological and hydrogeochemical features of the spring revealed that the hot spring water is of Na-HCO_3_ type. The spring water was inferred to be a mixture of shallow meteoric water (92%) and deep hydrothermal water (8%)^56^. A reservoir temperature of 220 °C had been estimated from geothermometers^76^.

### Geology and Seismotectonics

Geology at the monitoring site is characterized by Caledonian granite/gneiss and Cambrian sandstones. The outcropping bedrocks in the hydrothermal area are comprised of Middle Devonian dolomites, Cambrian quartz sandstone and feldspar sandstone, and Middle Proterozoic biotite-plagioclase granulite and migmatitic granite; overlain by Pliocene sandstone and gravelstone, and Holocene alluvium. Magmatic rocks including Himalayan andesite and basalt and Yanshanian granite were also found^76^. Tectonically, the Banglazhang hydrothermal area is located in the northeastern margin of the Indian-Eurasia plate collision zone, belonging to the Yunnan-Tibet geothermal belt in China, a major part of the Mediterranean-Himalayas geothermal belt. The springs occur on the EW-striking Xiangbai river sub-fault, which is bounded by the Longchuan fault to the northwest, the Longling-Ruili fault to the southeast, and the Nujiang fault to the east.

BLZ is located close to the epicentral area of the 1976 Longling *M*_W_ 7.0 earthquake (about 14 km from the earthquake’s epicenter; Fig. 1). Next to the epicentral area, 17 *M*_*S*_ ≥ 5.0 earthquakes (NEIC earthquake catalogs) have occurred since 1976, with 11 strong aftershocks in 1976. Following the earthquake series of 1976, various hydrothermal phenomena, such as hydrothermal explosions and geysers, have been observed in the Banglazhang hydrothermal area^77^.

### Observations and data

The BLZ hot spring monitoring site is maintained and operated by the China Earthquake Administration of Yunnan Province. Water from the spring is sampled once daily and measurements of radon have been performed routinely in a laboratory since 1976 April 6. The sample time is designated to occur at 8 o’clock in the morning in order to reduce the effect of daily variations. The radon concentration has been measured with three types of radon measurement instruments during the past 40 years. From 1976 April 6, to 1982 June 5, a FD-105 type radon gas detector was used, reporting the radon concentration in Eman. Eman is converted to the metric unit Bq/L using the relationship 1 Eman = 3.7 Bq/L. From 1982 June 6 to 2012 April 11, a FD-105K type electrometer (manufactured by Shanghai Electronic Instrument, co.) was used, the measurements given in Bq/L. Since 2012 April 12, a FD-125 type Radon & Thorium analyzer, manufactured by Beijing Nuclear Instrument Factory, sponsored by CNNC (China National Nuclear Corporation), has been used.

Water sampled from the spring is degassed by bubbling air and transported into a chamber, where the radon concentration is measured in a ZnS cell connected to a photomultiplier detector, and a scintillation counter. The measurement precision of the instruments is 0.1 Bq/L. A solid radium source (^226^Ra) with a known radioactive radon content is used for the calibration of the water radon under normal working conditions. This source is used to measure and calculate the calibration value of the instrument.

In addition to radon, water temperature and spring discharge rate are measured at the spring site when the water is sampled for radon. Temperature is measured using a mercury thermometer with a resolution of 0.1 °C. Discharge rate is measured using the stopwatch capacity method, i.e., the required time per unit volume of water is measured. Barometric pressure has been measured since 1997. Regional rainfall data were downloaded through the CPC Merged Analysis of Precipitation (CMAP) for the same period to evaluate its possible influence on radon in the present study.

GCRs are high energy particles (GeV and above) produced in our Galaxy by supernova explosions and by objects such as neutron stars and black holes. Emissions of matter and electromagnetic fields from the Sun increase during high solar activity, making it harder for GCRs to reach Earth, i.e. solar winds modulate GCR flux. Whereas solar irradiance varies of the order of 0.1% on decadal time scales, the intensity of cosmic rays varies globally by about 15%^51^. Both GCR and solar activity show a concurrent period of about 11 years. So GCR was selected as a proxy for solar activity for the comparison with radon. The hourly data of GCR monitored at McMurdo station were provided by Bartol Research Institute (refer to the supplementary materials). GCRs were measured by a 18-tube NM64-type neutron monitor that measures the number of high-energy particles impacting Earth from space.

To evaluate potentially earthquake-induced hydrological changes, the energy densities *e* (J/m^3^) of local and distant earthquakes were calculated according to eq. (6)^78^.

6 $$\mathrm{log}\,r=0.48M-0.33\,\mathrm{log}\,e-0.4$$

where *M* is the magnitude of the event and *r* is the epicentral distance in km.

Only those events above a threshold value of 10^−3^ J/m^3^ have been considered. The NEIC (National Earthquake Information Center, refer to the supplementary materials) earthquake catalog was used. In terms of seismic energy density, 84 earthquakes with energy densities larger than 10^−3^J/m^3^ were selected from between 1976 April to 2015 December (all with magnitudes greater than *M*_S_ 5.0, see Fig. 1 for their locations and Fig. S1d for their energy density-time history). The maximum energy density of 634 J/m^3^ was caused by the Longling *M*_W_7.0 earthquake of 1976 May 29, which occurred at a distance of 14 km from the monitoring site BLZ. The furthest earthquake is the 2011 *M*_W_ 9.0 Tohoku earthquake, with an energy density of 10^−2^ J/m^3^, which did not induce any obvious co-seismic water radon changes.

## Electronic supplementary material

Decadal radon cycles in a hot spring
