# Supplementary material for: Decadal radon cycles in a hot spring
Source: Sci Rep. 2017 Sep 21;7:12120. doi: 10.1038/s41598-017-12441-0 (PMC5608902; doi:10.1038/s41598-017-12441-0)
Supplement: Supplementary file 1 — Decadal radon cycles in a hot spring [file 41598_2017_12441_MOESM1_ESM.pdf]

Supplementary materials for

Decadal radon cycles in a hot spring

Rui Yan[a, b, c], Heiko Woith[d*], Rongjiang Wang[d], Guangcai Wang[a, b]

a State Key Laboratory of Biogeology and Environmental Geology&MOE Key Laboratory of Groundwater Circulation and Environmental Evolution,China University of Geosciences, Beijing 100083, China.

b School of Water Resources and Environment, China University of Geosciences, Beijing 100083, China.

c China Earthquake Networks Center, 100045 Beijing, China.

d GFZ German Research Centre for Geosciences, D-14473 Potsdam, Germany

This file contains (i) links to the data used in this study, and (ii) supplementary text and figures about the response of the hot spring to earthquakes, as well as details about radon solubility.

Data

The daily monitoring data from the hot spring will be made available as persistent electronic data supplements (with its own doi) through GFZ German Research Centre for Geosciences. An example is shown at http://gfzpublic.gfz-potsdam.de/pubman/faces/viewItemOverviewPage.jsp. Regional rainfall data were downloaded through CPC Merged Analysis of Precipitation (CMAP) (http://www.esrl.noaa.gov/psd/data/gridded/data.cmap.html). The hourly data of Galactic Cosmic Ray (GCR) monitored at McMurdo station, provided by Bartol Research Institute, were downloaded from http://neutronm.bartol.udel.edu/~pyle/bri_table.html. The NEIC (National Earthquake Information Center) earthquake catalog were downloaded from http://earthquake.usgs.gov/earthquakes/search/.

Response to earthquakes

Daily raw data of nearly 40 years of continuous monitoring are presented in Fig. S1. The concentration of radon dissolved in water ranged between 39.9 Bq/L and 1836 Bq/L, water temperature fluctuated between 41.9°C and 93°C, discharge rate varied between 0.0001 L/s and 0.02 L/s (Table S1). The wide range of parameters is due to a series of strong earthquakes which occurred next to the monitoring site in 1976.

***Table S1**. Descriptive statistics of the measurements at BLZ hot spring site: N - Number of daily samples; N_miss - Number of missing values; Max_gap - Maximum gap length in days; Min - minimum; Max- maximum; SD- standard deviation.*

Measurement	Start_date	N	N_miss	Max_gap	Min	Max	Median	Mean	SD
Radon (Bq/L)	1976 Apr. 6	14466	48	15	39.9	1836.0	249.0	265.0	143.2
	1979 Jan. 1	13481	33	15	40.7	696.0	242.0	246.0	101.3
Temperature (℃)	1976 Apr. 6	14479	17	2	41.9	93.0	85.8	85.4	3.5
	1979 Jan. 1	13503	11	2	71.4	93.0	85.8	85.5	3.3
Discharge rate (L/s)	1976 Jun. 16	14412	21	10	0.0001	0.02	0.011	0.0108	0.003
	1979 Jan. 1	13495	19	10	0.003	0.02	0.011	0.011	0.003

***Table S2.** Coefficient estimates of a linear regression and standard error. The coefficients a and b correspond to y=ax+b. Se_a and Se_b indicate standard errors of the coefficient estimates. RMSE means root mean square error.*

Measurement	a	Se_a	b	Se_b	RMSE
Radon (Bq/L)	-2.04E-03	1.19E-03	1732.59	871.19	98.16
Temperature (℃)	-4.09E-04	3.41E-05	384.20	24.89	2.80
Discharge rate (L/s)	-6.10E-07	2.22E-08	0.46	0.02	0.0018
GCR (counts/hour/100)	7.95E-02	6.99E-03	-48725.64	5102.15	574.88
Atmosphere pressure (hPa)	-1.06E-04	9.97E-05	948.54	73.04	3.01
Rainfall (mm/day)	-1.40E-05	4.28E-05	13.73	31.20	3.52
Monsoon Rainfall (mm/day)	-2.83E-02	1.36E-02	64.56	27.07	0.88

Table S3 *Variations of hydrogeological observations caused by earthquakes: Lat, Lon - latitude and longitude of earthquake; Mag, depth - magnitude and depth of earthquake; Azm, Dis - azimuth and distance to the epicenter; ED - energy density.*

Date (yyyy/mm/dd)	HH:MM:SS (UTC)	Lat (°)	Lon (°)	Mag (*M*)	Depth (km)	Azm (°)	Dis (km)	ED (J/m^3)	Radon (Bq/L)	Temperature (℃)	Discharge (L/s)
1976/05/29	12:23:18	24.57	98.95	6.9	8	108	29	48.661	359	-5.5	No measurement
	14:00:18	24.53	98.71	7.0	10	166	14	634.194			
	19:36:55	24.55	98.93	5.2	32	114	28	0.184			
1976/05/30	04:18:43	24.42	98.81	5.1	28	152	29	0.118			
1976/05/31	05:08:28	24.34	98.64	6.2	14	186	35	2.728			
	18:35:05	24.38	98.77	5.5	20	162	32	0.349			
1976/06/09	00:20:39	24.89	98.75	5.9	33	16	28	2.038	757.4	-6.5	
1976/07/21	15:10:45	24.78	98.70	6.3	9	10	14	54.611	1273	-41.1	-0.00781
1976/07/23	01:43:58	24.89	98.68	5.0	33	1	27	0.112			
1976/10/12	15:19:33	24.48	98.81	5.0	33	145	23	0.163	132	4.1	-0.00167
1996/02/03	11:14:20	27.29	100.28	6.6	11	29	334	0.011	188	-1.4	-0.00130
2004/12/26	00:58:53	3.30	95.98	9.1	30	187	2392	0.123	212.7	-4.3	-0.00313
2005/03/28	16:09:36	2.09	97.11	8.6	30	184	2514	0.020	90	No response	No response

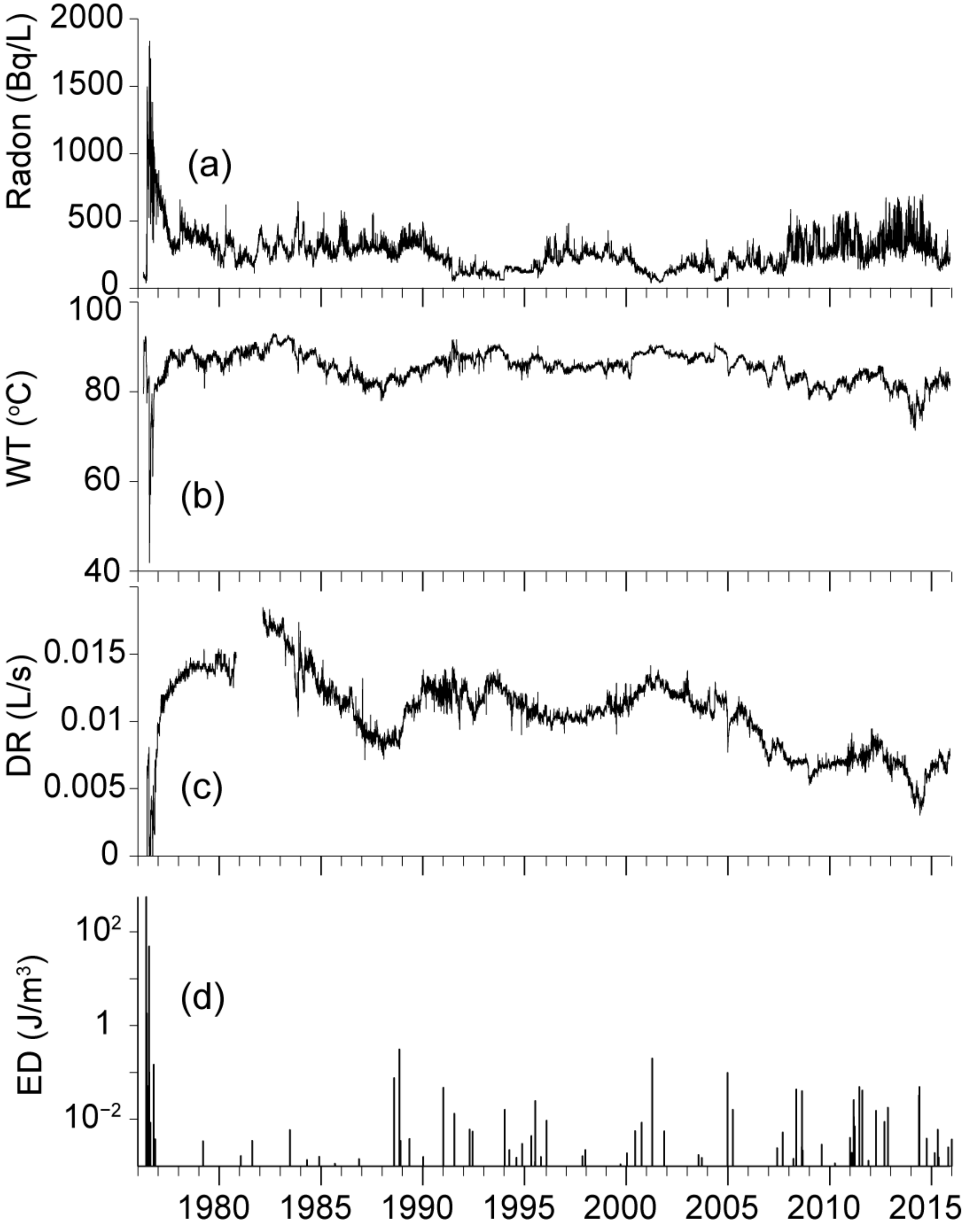

Fig. S1. *Time series of daily groundwater parameters at the BLZ hot spring site, from top to bottom panels: (a) Radon, (b) Water Temperature WT, (c) Discharge rate DR, (d) Earthquakes with energy density ED larger than* 10^{-3} *J/m*^{3}.

In order to identify hydrological response to earthquakes, 84 earthquakes with energy density > 10^{-3} J/m^3 at BLZ hot spring site were examined. For each of the 84 earthquakes

it was checked whether coseismic radon changes were induced by the earthquakes after taking the influence of precipitation into account. Only the earthquakes listed in table S2 induced apparent coseismic radon changes. Additional to the series of events in 1976, hydrological changes induced by earthquakes were identified following seismic events in 1996, 2004, and 2005. Totally, the events with energy density > 0.1 J/m^3 at BLZ can induce observably hydrological changes at BLZ site. While 17 out of 19 events with energy density from 0.01 to 0.1 J/m^3 did not induce obvious hydrological change at BLZ site. For example, the Wenchuan 2008 M_W7.9 earthquake with energy density of ~0.05 J/m^3 also did not induce obvious changes in the hydrological parameters of BLZ site.

Extremely large changes were caused by the 1976 Longling M_W7.0 earthquake and its aftershocks (see Fig. S1-2): Radon increased co- and postseismically from about 50 Bq/L to 1,800 Bq/L. The increase was not continuous, but occurred as a sequence of step-like increases and decays in relation to the main shock and the following aftershocks. The radon increases were accompanied by drops in the water temperature. The largest changes in all parameters were not related to the main shock, but to the M=6.3 event in July 1976: radon increased by 1,273 Bq/L, temperature dropped by 41°C, and the spring discharge (measurements started in June 1976, i.e. no data available for the main event) dropped from 0.008 L/s to almost zero.

BLZ is located in the near-field of the 1976 earthquake series (about 14 km from the major earthquake epicenter). Thus, both static and dynamic pore pressure variations were expected to be significant. We used the computer code “tfcmb” (developed by one of the co-authors R. Wang; the program is freely available from the author upon request) based on the elastic dislocation model [1] to calculate the static co-seismic volumetric strain changes under un-drained conditions. Pore pressure changes are assumed to relate to the confined pressure with the Skempton parameter. The Poisson ratio was set to 0.25, the shear modulus to 30GPa, the Skempton parameter to 0.8, and the friction coefficient to 0.7. Harvard CMT solutions were used as input parameters. The two events which both occurred on 1976 May 29 had by far the largest impact on BLZ: The pore pressure dropped by 0.33 bar and 0.50 bar following the M_w=6.6 event at 12:23:29 and the M_w=6.6 at 14:00:33, respectively. The corresponding volume strain changes are 8.27×10^{-07} and 1.24×10^{-06}. The M_w=6.1 event of 1976 May 31 had a minor effect at BLZ: The pore

pressure dropped by 0.07 bar corresponding to a static strain change of 1.78×10^{-07}. The same applies for the M_W=6.1 event of 1976 July 21 which caused a pore pressure increase of 0.07 bar corresponding to a static strain change of -1.69×10^{-07}. Thus, static pore pressure changes likely do not explain the observations. It is fair to note, that the modelling results strongly depend on the location of the ruptured faults with respect to the monitoring site.

Qualitatively, the same behavior had been observed after the Lijiang M_W 6.6 earthquake of 1996 (Fig. S3) and the 2004 Sumatra M_W 9.1 earthquake (Fig. S4). A summary of all induced variations is given in table S2. From the timeseries presented in Fig. S2-S4 it is evident that the response is relatively fast (days to weeks), while the recovery to the pre-earthquake values is slow, indicating that hydrogeological parameters recovered to background levels within one year.

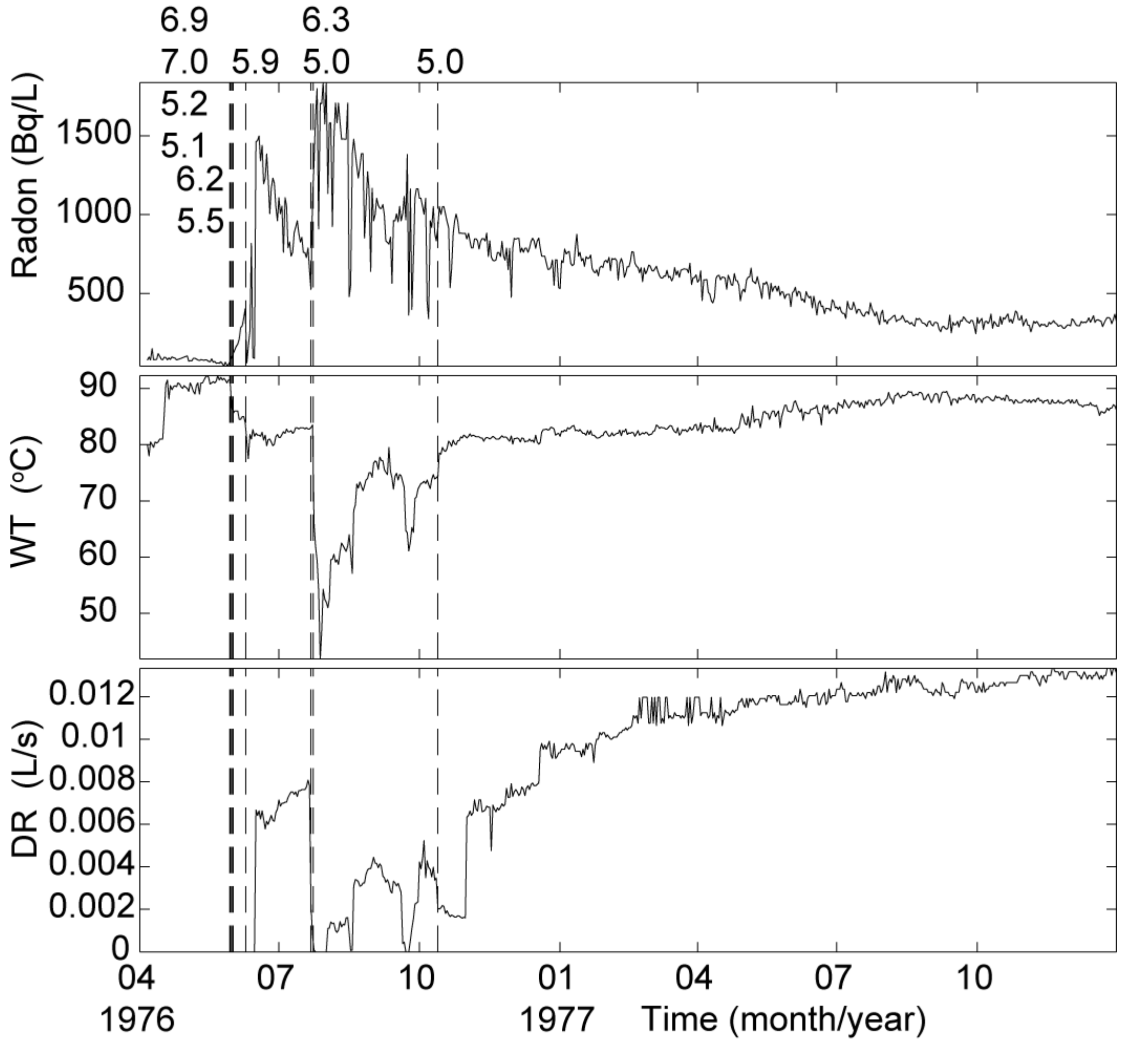

Fig. S2. *Changes and recovery processes of radon, water temperature WT, and discharge rate DR caused by the 1976*

Longling Mw7.0 earthquakes series. Detailed information about the earthquakes is listed in table S3. Radon and temperature were observed since 1976 April 6, discharge rate measurements started 1976 June 16.

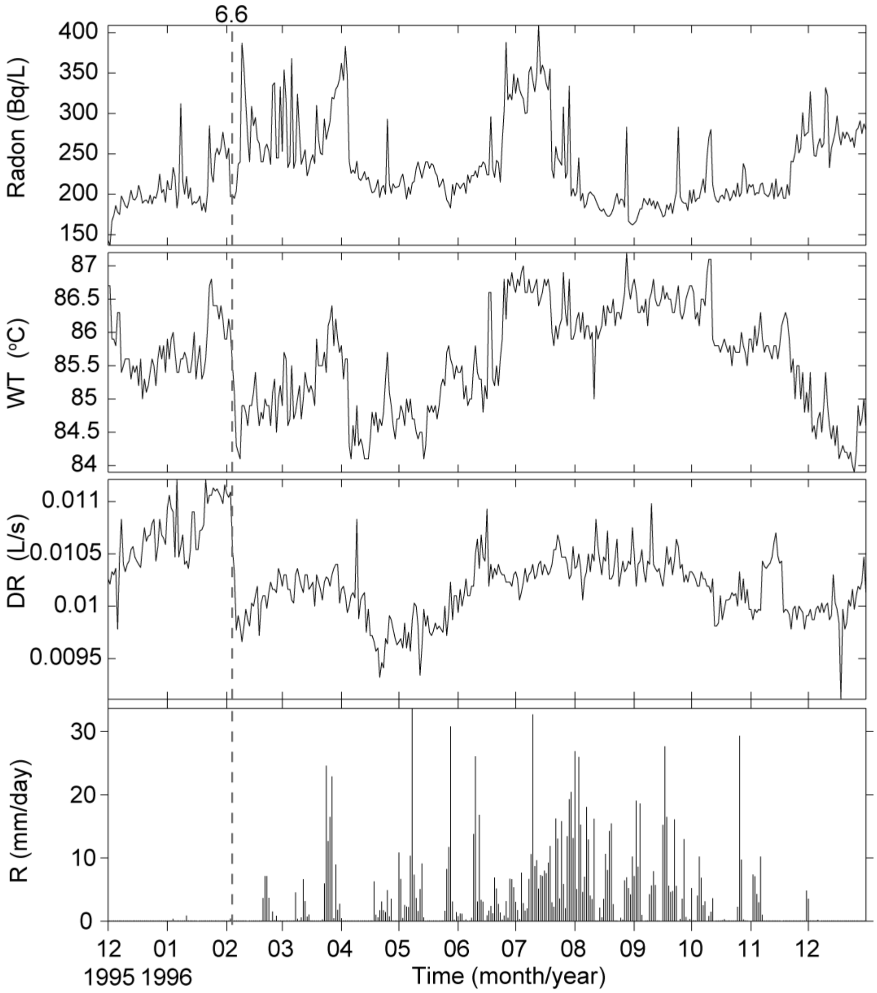

Fig. S3. *Changes and recovery processes of radon, water temperature, and discharge rate caused by the M=6.6 earthquake of 1996 February 3 which occurred at a distance of 334 km. Rain fall is shown for comparing the effect of earthquake.*

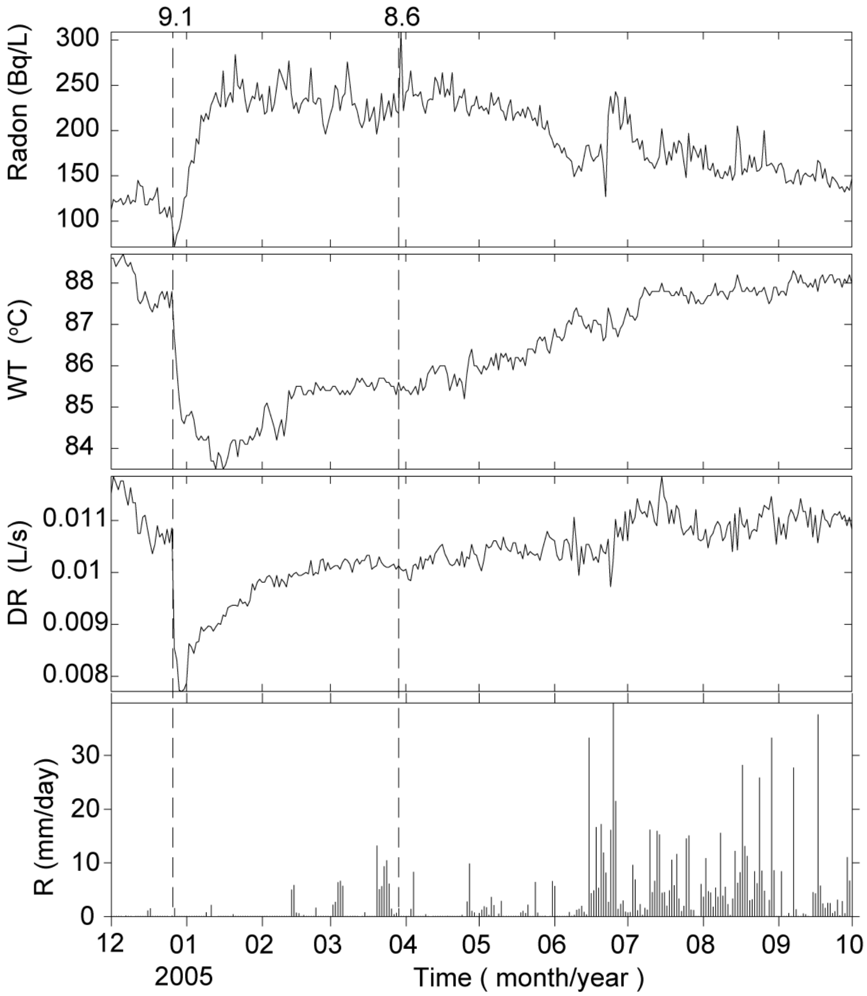

Fig. S4. *Changes and recovery processes of radon, water temperature, and discharge rate caused by the 2004 Sumatra M_W 9.1 and the 2005 Sumatra M_W 8.6 earthquakes. Detailed information about the earthquakes is listed in table S3. Rain fall is shown for comparing the effect of earthquake.*

Radon solubility

The solubility of a gas in a liquid is temperature-dependent and proportional to the partial pressure of the gas. The solubility coefficient S for radon can be estimated from an empirically derived formula (see e.g. Koike et al.[2], for further discussions)

$$S = 0.1057 + 0.405 \cdot \exp(-0.0502 \cdot T)$$

The tabulated solubilities usually refer to normal pressure conditions (1013.25 hPa), but the average barometric pressure at BLZ is 870 hPa. Taking the water vapour pressure at the BLZ altitude (1,280 m a.s.l.) into account, the actual radon solubilities at BLZ are even lower, i.e. 14% less radon can be kept in solution compared to sea-level. The quasi-decadal maxima and minima of the water temperature are 93°C and 71°C, corresponding to radon

solubility coefficients of 0.092 and 0.099, respectively (Fig. S5). Thus, at 71°C about 0.7% more radon can be dissolved in water as compared to 93°C. At the same time, radon concentrations varied from 41 Bq/L during high-temperature periods up to 696 Bq/L during times of low water temperatures. The large radon range likely cannot be explained by temperature-dependent solubility alone.

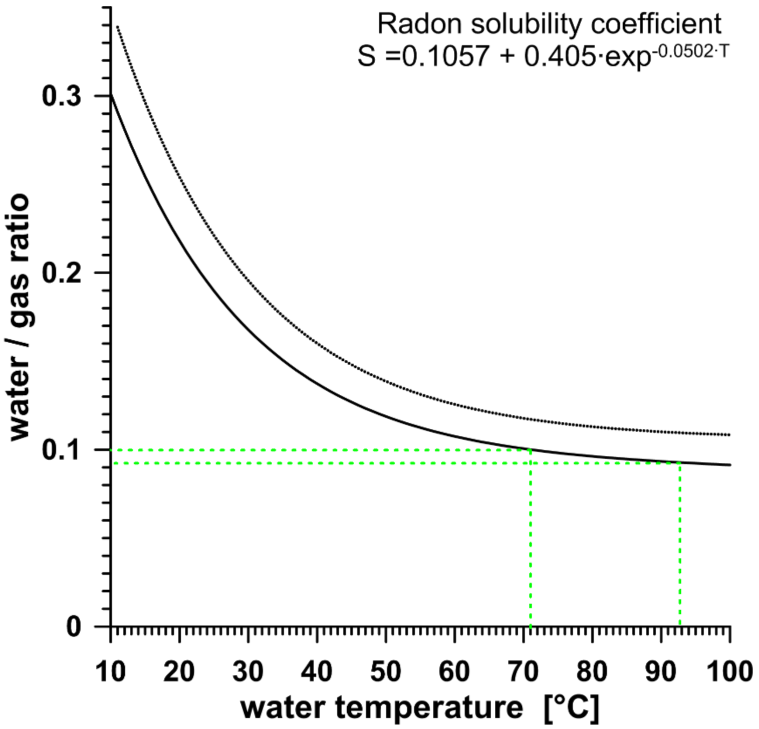

Fig. S5 *Radon solubility as a function of temperature T. The black dashed line refers to sea-level conditions (1013.25 hPa), whereas the black line refers to the average barometric pressure of 870 hPa at the altitude of BLZ (1280 m). Green dotted lines indicate the decadal maxima (93°C) and minima (71°C) water temperatures at BLZ, respectively.*

References

1 Okada, Y. Internal deformation due to shear and tensile faults in a half-space. *Bulletin of the Seismological Society of America* **82**, 1018-1040 (1992).

2 Koike, K., Yoshinaga, T. & Asaue, H. Characterizing long-term radon concentration changes in a geothermal area for correlation with volcanic earthquakes and reservoir temperatures: A case study from Mt. Aso, southwestern Japan. *Journal of Volcanology and Geothermal Research* **275**, 85-102, doi:http://dx.doi.org/10.1016/j.jvolgeores.2014.02.007 (2014).
